# An interactive tool to forecast US hospital needs in the coronavirus 2019 pandemic

**DOI:** 10.1093/jamiaopen/ooaa045

**Published:** 2020-11-30

**Authors:** Kenneth J Locey, Thomas A Webb, Jawad Khan, Anuja K Antony, Bala Hota

**Affiliations:** 1 Center for Quality, Safety and Value Analytics, Rush University Medical Center, Chicago, Illinois, USA; 2 Knowledge Management Services, Rush University Medical Center, Chicago, Illinois, USA; 3 Division of Plastic and Reconstructive Surgery, Department of Surgery, Rush University Medical Center, Chicago, Illinois, USA; 4 Division of Infectious Diseases, Department of Internal Medicine, Rush Medical College, Chicago, Illinois, USA

**Keywords:** COVID-19, epidemiology, healthcare, forecasting, modeling, SEIR

## Abstract

**Objective:**

We developed an application (https://rush-covid19.herokuapp.com/) to aid US hospitals in planning their response to the ongoing Coronavirus Disease 2019 (COVID-19) pandemic.

**Materials and Methods:**

Our application forecasts hospital visits, admits, discharges, and needs for hospital beds, ventilators, and personal protective equipment by coupling COVID-19 predictions to models of time lags, patient carry-over, and length-of-stay. Users can choose from 7 COVID-19 models, customize 23 parameters, examine trends in testing and hospitalization, and download forecast data.

**Results:**

Our application accurately predicts the spread of COVID-19 across states and territories. Its hospital-level forecasts are in continuous use by our home institution and others.

**Discussion:**

Our application is versatile, easy-to-use, and can help hospitals plan their response to the changing dynamics of COVID-19, while providing a platform for deeper study.

**Conclusion:**

Empowering healthcare responses to COVID-19 is as crucial as understanding the epidemiology of the disease. Our application will continue to evolve to meet this need.

## LAY SUMMARY

Hospitals have been continually faced with anticipating the resurgent spread of COVID-19 and its effects on visits, admissions, bed needs, and crucial supplies. However, few open-source tools are available to aid hospitals in planning. We developed a web application (https://rush-covid19.herokuapp.com/) for US states and territories to predict the spread of COVID-19 and to provide forecasts for hospital visits, admissions, discharges and to anticipate needs for intensive care unit (ICU) and non-ICU beds, ventilators, and personal protective equipment. Users can choose from a suite of models to predict the spread of COVID-19, some of which explain >99% of variation in COVID-19 cases within states. Users can modify a large set of inputs to obtain forecasts for their institution, examine variability in forecasts over time, download forecast data for further analysis, and explore trends in hospitalization and testing. We designed our application to be interactive, insightful, and easy to use for hospital leaders, healthcare workers, and government officials. However, specialists can use our models, open-source code, and aggregated data for deeper study. As the dynamics of COVID-19 change, our application will also change to meet emerging needs of the healthcare community.

## INTRODUCTION

Healthcare enterprises are grappling with the challenges of preparing for the resurgence of Coronavirus Disease 2019 (COVID-19) and appropriation of resources needed to treat patients while protecting healthcare workers. We developed an open-source application that generates hospital-specific forecasts of COVID-19 patient needs and related supplies. Our application (https://rush-covid19.herokuapp.com/) allows users to employ a suite of models to predict the spread of COVID-19 and to generate forecasts for COVID-19 visits, admits, discharges, and needs for intensive care unit (ICU) and non-ICU beds, ventilators, and personal protective equipment (PPE). Users can also examine trends in hospitalization and testing.

We describe our application in detail, focusing on the data and models it uses, interactive inputs, and provided forecasts. We explain the use of our application and the caveats of our approach and discuss its value in aiding the needs of the healthcare community and its potential for generating novel insights. Our efforts were primarily aimed at addressing urgent healthcare demands and enabling informed decision-making. Our application uses open-source software and it source code and aggregated data are publicly available.

## DATA, MODELING, AND USE

### Application development

We developed our application in response to the needs of our home institution—Rush University System for Health—in anticipation of COVID-19 cases and subsequent surges. A COVID-19 command center staffed by senior hospital administrators served as a real-time focus group for initial requirements and choice of user-defined variables. Feedback from the Illinois Hospital Association hospitals aided further refinement.

### Data

Our application aggregates reports of cumulative cases across US states and territories from the Johns Hopkins University Center for Systems Science and Engineering repository,^1^ state and territory population sizes based on US Census Bureau data (2010–2019), dates of COVID-19 arrival from state and territory health agencies, and testing and hospitalization levels from The Atlantic’s COVID Tracking Project.^2^

### Forecasting cumulative COVID-19 cases

We developed 7 models to forecast COVID-19 case volumes up to 60 days past the present day.

### Exponential growth

Initial spread of infection may be limited primarily by inherent growth rate (*r*), which proceeds multiplicatively according to the function, *N_t_* = *N_0_e^rt^*. Here, *N_0_* is the initial infected population size, *t* is the change in time, and *N_t_* is the infected population size at *t*. This model was widely used to characterize the spread of COVID-19 during initial weeks of infection.^3–5^ Our application uses a regression on the log-linear transformation, log(*N_t_*) = log(*N_0_*) + *t* · *r*, to predict the expected number (*N*) of cumulative cases.

### Quadratic growth

**Figure 1.**
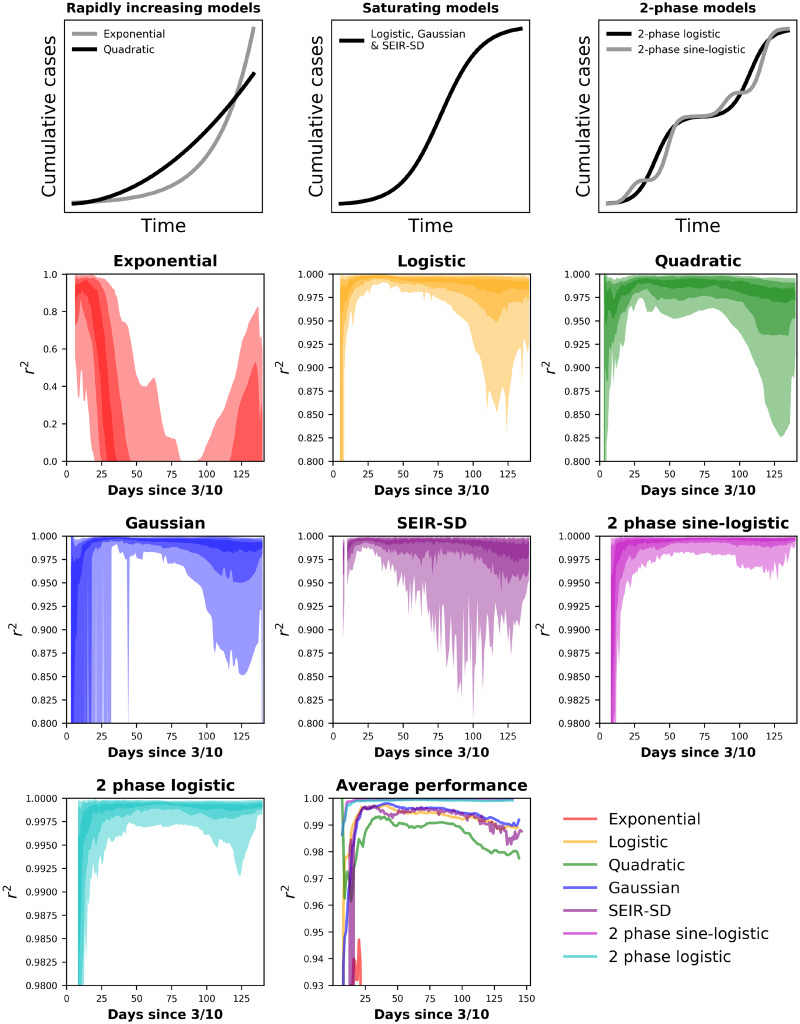
Top row: General forms of our application’s models. Rows 2–4: 95, 75, and 65% confidence interval (CI) hulls for the performance of models across US states and territories from March 16 to August 1. The Johns Hopkins University Center for Systems Science and Engineering data shows most states beginning to report COVID-19 cases by March 10 and we allowed the models a week of data before fitting them. The *x*-axis is in days since the first reported case, occurring on or after March 10. Lightly colored hulls are 95% CI, hulls of intermediate darkness are 75%, and the darkest hulls are 65% CI. Performance is measured via a modified *r*^2^ of observed versus predicted values where the *y*-intercept is forced through 0.^10^ The *y*-axes are scaled to show the greatest resolution for each model and are not scaled similarly across all models. Lower right: Mean *r*^2^ values across states for all models.

Initial growth may be more rapid than expected from the exponential model and also characterized by a constant change in growth rate (Figure 1). In this case, growth may be quadratic. Early COVID-19 studies implicated quadratic growth of COVID-19.^6^^,^^7^ The quadratic function is a second-order polynomial that applies to population growth as *N_t_* = *β*_1_*t*^2^ + *β*_2_*t* + *N_0_*. Our application predicts values for (*N*) using numerical optimization of *β*_1_ and *β*_2_. Like the exponential model, this model does not account for eventual leveling-off.

### Logistic growth

The spread of a contagious disease must eventually slow as limitations are encountered (eg, immunity). The logistic model captures this dynamic and produces an *s*-shaped curve^8^^,^^9^ (Figure 1). Early COVID-19 studies implicated logistic growth in the spread of the disease.^11^^,^^12^ The logistic model takes a simple functional form, *N_t_* = *α*/1 + *e*^−^^*rt*^, where *α* is the upper limit of *N* and *r* is the intrinsic rate of increase. Our application uses numerical optimization of *α* and *r* to find the best fit logistic function and predicted values for *N_t_*.

### Gaussian growth

The Gaussian (ie, normal) distribution can provide a fast approximation to some epidemiological models.^13^ The Gaussian distribution has 2 parameters, mean (*μ*), standard deviation (*σ*) (Figure 1). When used to model the spread of disease, Gaussian curves are symmetrical around a climax day. Gaussian models have successful in approximating the spread of COVID-19.^14^ Our application finds the best fit cumulative Gaussian function via numerical optimization of *μ* and *σ*.

### SEIR-SD

COVID-19 modeling has often used refinements to the SEIR model,^4^^,^^15–18^ which accounts for changes in fractions of susceptible persons (*S*), persons exposed but not exhibiting infection (*E*), infectious persons (*I*), and those recovered (*R*), where *S *+* E* + *I *+* R *=* *1. These subpopulations are modeled in a set of differential equations:

$$\frac{dS}{dt}=\;\beta SI,\;\frac{dE}{dt}=\beta SI-\alpha E,\;\frac{dI}{dt}=\alpha E-\gamma I,\;\frac{dR}{dt}=\gamma I.$$

Here, *α* is the inverse of the latent period, *γ* is the inverse of the mean infectious period, and *β* is the mean number of effective contacts (ie, contacts resulting in secondary infection). Our application imputes the initial value of *β* from a relationship between *γ* and the basic reproductive number (*R_0_*), that is, *β = γR_0_* (Table 1).^26–28^ It then allows *β* to decrease in proportion to *I*, from which an implicit frequency-dependent degree social distancing emerges. Our model also simulates social distancing (*λ*) as an explicit response to public policy by including a time-iterative modification to *β*, that is, *β_t_*_+1_ = *β_t_*/(*λI* + 1), where *β* remains unchanged when *I* or *λ* equal 0. When *λ* equals 1, the daily change in *β* is governed by the effect of *I*. The product of *I* and *λ* then determines the daily change in *β*: *λI* = (*β_t_* − *β_t_*_+1_)/*β_t_*_+1_. Finally, our model captures lags in testing by allowing the apparent size of *I* to lag behind the actual magnitude via use of a logistic function: *I_t_*/1+e^−^^*f*^^(t)^, where *f*(*t*) ∼ *τt*.

**Table 1.** Parameters for the SEIR-SD model

Parameter	Description	Reported range	References
Average latent period	Days during which an exposed individual cannot infect others	5–6 days	^18–22^
Average infectious period	Days over which a person remains infective	1.6–13 days	^18^ ^,^ ^23–25^
Basic reproductive number (*R_0_*)	Average number of secondary infections generated by a primary infection in a totally susceptible population	2.1–6.5	^4^ ^,^ ^18^ ^,^ ^20^ ^,^ ^23^ ^,^ ^24^

*Note:* Our application attempts to optimize the following SEIR-SD parameters within ranges of reported values for the average latent period, the average infectious period, and the basic reproductive number.

Our application performs 200 000 iterations on combinations of SEIR parameter values, within reported ranges (Table 1). It then chooses the set of parameters that maximize the explained variation in observed data, which avoids the challenges of applying numerical optimizers to complex models.

### Two-phase logistic growth

Many US states have experienced a resurgence in COVID-19, leading to increases that simple models and classic epidemiological models fail to capture. To capture resurgence, we expanded the logistic model to include 2 phases. Using the functional form of the logistic model, our application iteratively searches across time series to find the point where 2 phases of logistic growth best explain the variation in cumulative cases.

### Two-phase sine-logistic growth

An extension of the two-phase logistic, this model assumes periodic fluctuations in the increase of cumulative cases by including sine-wave dynamics to the logistic equation: *N_t_* = *α*/1 + *e*^−^^*rf*^^(^^*t*^^)^, where *f*(*t*) ∼ *t* + sine(*t*). As with the two-phase logistic model, our application searches across the time series to find the point where 2 phases of growth best explain variation in observed data.

### Forecasting hospital visits and admits via time lags

**Figure 2.**
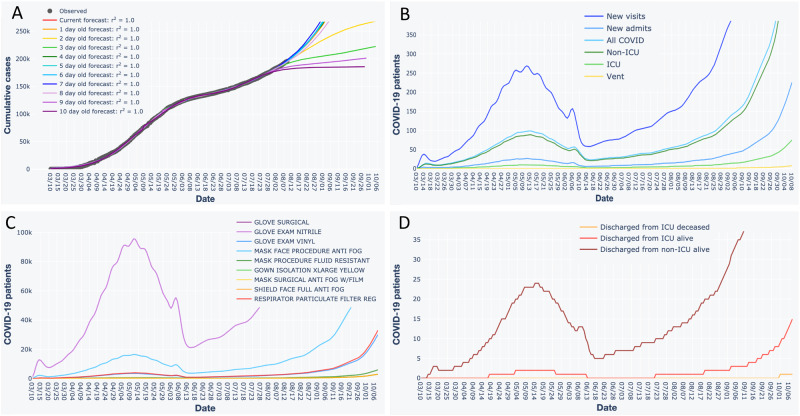
Interactive forecast plots generated by our application, using fits of the two-phase sine-logistic model to Illinois data as an example. (A) Forecast for cumulative COVID-19 cases. Black dots are observed data. Colored lines are forecasts. Values for coefficients of determination (*r*^2^) pertain to observed versus predicted values, where the *y*-intercept is forced through 0.^10^ (B) Forecasted patient census. (C) Forecasted personal protective equipment supply needs. (D) Forecasted discharges. When using the application, users can pan across, zoom in, select and de-select data, hover over data points and lines for additional information, and download plots as a png files. The application also presents information in B–D as tables, which users can download as csv files.

Users can obtain interactive forecasts for hospital visits and admists by entering expected daily values for percent of state-wide COVID-19 cases visiting their hospital, percent of those admitted, and the number of transfer patients accepted (Figure 2). We accounted for the tendency of infected persons to not seek immediate attention by modeling time lags as Poisson random variables, whereby newly infected persons wait 0, 1, etc., days to visit the hospital. The Poisson model is a discrete probability distribution where the mean corresponds to the expected average time lag. This approach avoided overburdening users by not requiring parameter values for which data may not be readily available.

**Figure 3.**
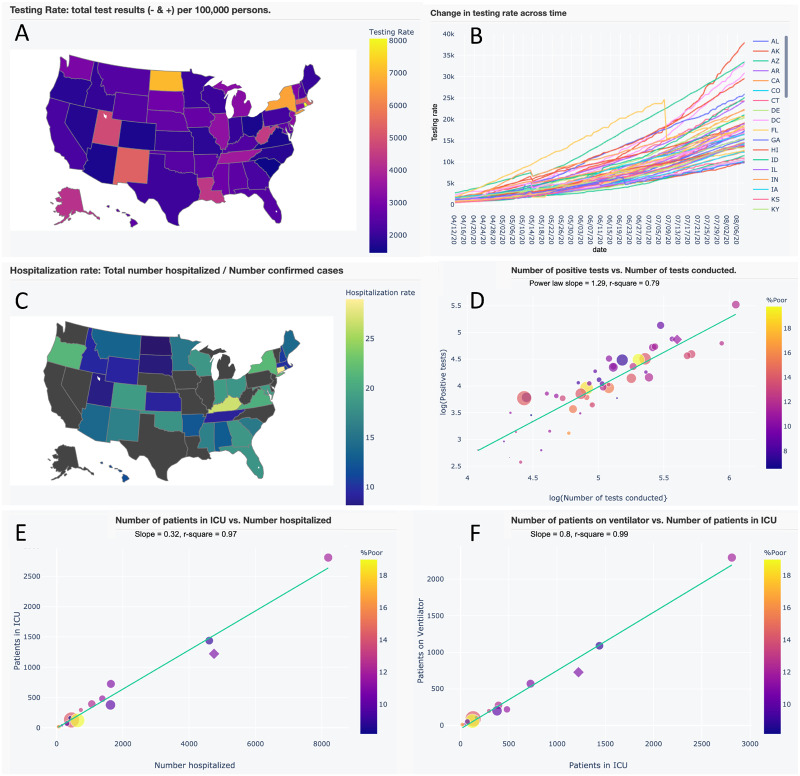
A subset of interactive maps and statistical relationships from our application’s Trends in Hospitalization and Trend in Testing tabs. Users can pan across, zoom in, select and de-select data, and hover over data for additional information. (A) Map of testing rate (tests per 100 000 persons). (B) Time series of testing rates across US states and territories. (C) Map of hospitalization rate (total number of persons hospitalized/no. of confirmed cases). (D) A strong power-law (*y* ∼ *x^z^*) relationship between positive test results and number of tests conducted, where the slope (*z*) indicates that greater testing reveals disproportionately greater positive results. (E and F) Strong linear relationships between the number of patients in intensive care unit (ICU) versus the number hospitalized and the number of patients on ventilators versus those in ICU. These latter relationships may be used to aid in parameterizing forecast variables.

### Forecasting hospital bed, ICU needs, and discharges via daily carry-over

Users can obtain interactive forecasts for hospital bed, ICU needs, and discharges by entering parameter values for the number of ICU and non-ICU beds in house and choose expected values for the percent of COVID-19 patients admitted to ICU, average length of stay (LOS) for ICU and non-ICU patients, percent of ICU patients on ventilators, and ICU mortality (Figure 2). These inputs drive calculations for newly admitted ICU, non-ICU, and ICU-ventilator patients expected each day, as well as numbers of discharges. Our application models the daily patient carry-over using a binomial model, a probability distribution for binary outcomes (eg, patients leave or stay for an additional day) that requires 2 parameters (*p*, *n*). We set the value of *p* to 0.5 and set the value of *n* to be twice the LOS, which produces a probability mass function (pmf) with a mean equal to the LOS. This pmf is converted to a cumulative distribution function to obtain estimates for the fraction of 1-day, 2-day, etc., patients discharged on the present day. Patients not discharged are iteratively carried over to the following days. Like the Poisson, the binomial does not overburden the user with parameters based on data they may not readily have.

### Forecasting supply needs

Users can obtain forecasts for PPE needs after entering expected per patient daily values for surgical gloves, nitrile exam gloves, vinyl exam gloves, anti-fog procedural face masks, fluid resistant procedural face masks, extra-large yellow isolation gowns, anti-fog surgical face masks, anti-fog full face shields, and particulate filter respirators (Figure 2). Multiplying the expected PPE values by their respective patient type across the forecasted census produces PPE forecasts.

### Downloadable forecast data

Users can download data pertaining to each forecast. These csv files are dynamically updated upon any changes to their associated graphs or tables.

### Source code and Data

We developed our application using open-source software (python 3.7, plotly dash) and the Heroku web-hosting service. Source code, data, and README file for our application and for reproducing analyses herein are available at https://github.com/Rush-Quality-Analytics/SupplyDemand and via the Dryad Digital Repository at https://doi.org/10.5061/dryad.1ns1rn8rx.^29^

## RESULTS

### COVID-19 predictions

The exponential model explained >90% of variation in cases during initial weeks of infection but quickly began to fail (Figure 1). Its performance then increased with the onset of resurgence. The logistic, quadratic, Gaussian, and SEIR-SD models explained >95% of variation in cases among states but suffered with the onset of resurgence (Figure 1). Because of their design to capture resurgence, the two-phase logistic and sine-logistic models generally explained >99% of variation in cases (Figure 1).

### Use upon release

Our application has been in continuous use by our home institution to forecast our patient census and PPE needs or to examine observed trends against forecasts. Our application was also used on a daily basis by a NY hospital, whose frequent feedback led to several modifications.

## DISCUSSION

We expect forecasting tools to continue informing operational responses until a vaccine is available and the spread of COVID-19 subsides. Because we intend for the usefulness of our application to extend throughout the pandemic, we recently implemented accurate resurgence models, analyses of testing and hospitalization, and numerous modifications from user feedback. Because different hospitals in different states may experience resurgence at different times, the demand for our application at individual hospitals will vary as local areas experience peaks and valleys in COVID-19 infections.

Our application also provides insights beyond its primary use. First, few SEIR models account for social distancing as a combination of an emergent social response and public policy, or account for time lags in testing. Second, our two-phase resurgence models offer novel extensions of the popular logistic model. Both can be extended to any number of phases and the two-phase sine-logistic represents a novel combination of logistic growth, resurgence, and periodic fluctuations. Third, our application revealed strong statistical relationships in testing and hospitalization that have otherwise received little attention (Figure 3). Finally, researchers could conduct future studies using the downloadable data from our application, its aggregated source data, or by modifying the source code.

Hospitals should weigh the accuracy of our tool against the knowledge of their system’s needs to avoid over/under allocation of resources. The following should also be considered when using our application: first, we used a popular COVID-19 dataset that may not reflect true prevalence; second, values of input parameters such as LOS and PPE needs likely vary across time; third, LOS may not necessarily be binomially distributed and time lags in hospital visits may not necessarily be Poisson distributed; finally, our application does not account for differences in susceptibility to COVID-19 with respect to age, comorbidities, and sociodemographic factors.

## CONCLUSION

The worst pandemic in a century has coincided with a revolution in open-source tools and data science and has been met with numerous public-facing applications. Continued efforts should leverage a variety of models and deliver actionable predictions to prevent over-reliance on a single model and to couple the regional spread of COVID-19 to local healthcare needs.

## AUTHOR CONTRIBUTIONS

KJL developed the application’s models and source code, was responsible for deploying and maintaining the application, and wrote the manuscript. TAW guided application development and deployment and wrote the manuscript. JK assisted with website architecture and assisted with writing the manuscript. AKA provided input on the modeling and assisted with writing the manuscript. BH guided application development and wrote the manuscript.
